# Mitigating the effects of group negative affectivity on team creativity: the role of autonomy climate and leaders’ emotion management behavior

**DOI:** 10.3389/fpsyg.2026.1814428

**Published:** 2026-05-22

**Authors:** Moon Joung Kim, Jin Nam Choi

**Affiliations:** 1 Department of Business Administration, Cheongju University, Cheongju, Republic of Korea; 2 Department of Business Administration, Seoul National University, Seoul, Republic of Korea

**Keywords:** climate for autonomy, group negative affectivity, leader emotion management behavior, self-determination theory (SDT), team creativity

## Abstract

Drawing on conservation of resources (COR) theory and self-determination theory (SDT), this study examines how group negative affectivity that reflects the compositional input based on team members’ affective dispositions is related to team creativity. We further propose that leader emotion management behavior serves as a compensatory resource that may buffer this association. Using multi-source field data from 66 work teams (*N* = 293 employees and 66 managers), we find that group negative affectivity is negatively related to climate for autonomy, which is positively associated with team creativity. Our analysis also shows that this negative indirect relationship between group negative affectivity and team creativity via climate for autonomy is attenuated when leaders engage in emotion management behaviors for team members. These empirical patterns remained robust under a multilevel specification accounting for the nested structure of the data. Our findings highlight that emotionally responsive leadership plays a critical role in mitigating the negative implications of members’ negative affectivity and sustaining autonomy-supportive climates for team creativity.

## Introduction

Team creativity is becoming a vital component of organizational success, particularly in contemporary organizations where work is increasingly organized around teams. It refers to the collective generation of novel and useful outcomes by team members, underscoring the inherently collaborative nature of creative performance in organizations (Yuan et al., 2022). Thus, scholars have increasingly emphasized the interaction processes through which team members exchange, integrate, and develop ideas together (Barrett et al., 2021). Because these processes involve continuous communication, coordination, and interpretation among members, they are inherently affect-laden (Barsade and Gibson, 2007; Madrid et al., 2016). Accordingly, a substantial body of research has highlighted the importance of affective processes in shaping creativity-related outcomes, such as cognitive flexibility, motivation, and interpersonal dynamics (To et al., 2017). We enrich this literature by exploring the possibility that group-level affective inputs based on members’ affective dispositions predict group climate and team creative performance.

At the team level, prior research has primarily focused on group-level emotional states shared among members that arise through interpersonal interactions (e.g., Barsade and Knight, 2015; Knight and Eisenkraft, 2015). These approaches provide important insight into how shared emotions influence team functioning. However, they largely emphasize transient, interaction-driven states, thereby overlooking a more stable and enduring source of affective experiences of team members, that is, their dispositional, trait affectivity.

To address this critical omission, the present study shifts attention to group affective composition that reflects the stable dispositional affectivity of team members (Barsade and Knight, 2015). In contrast to group affective states, which capture short-term emotional experiences or moods of members shaped by situational cues and social interactions, group affective composition reflects relatively stable affective orientations of members. Similar to demographic characteristics or personality traits, these dispositional tendencies can systematically influence how members interpret and respond to task and social interactions within the team (Hood et al., 2016). With this focus on trait affectivity of members, the present study complements and extends prior work on group affective states or tones.

In the present study, we focus on group negative affectivity, defined as the average dispositional tendency of team members to experience negative emotions, such as anxiety, frustration, and hostility (Watson and Clark, 1984). Negative affectivity warrants particular attention because its effects on social interactions are often asymmetric and more pronounced than those of positive affectivity (Chi and Lam, 2022). Teams composed of members high in negative trait affectivity are more likely to exhibit defensive communication, emotional suppression, and conflict-laden interactions (Barsade and Gibson, 2007). These detrimental interaction patterns can impair members’ willingness to initiate and explore new approaches, thereby constraining the team’s capacity to produce creative outcomes (Reiter-Palmon and Paulus, 2019).

The multilevel emergence framework suggests that team-level phenomena do not arise directly at the collective level but emerge as members’ own affect, cognition, and motivation are amplified through interaction and manifested as shared collective properties (Kozlowski, 2025). From this perspective, compositional inputs such as affective traits do not directly determine team-level outcomes; rather, they shape recurring interaction patterns through which shared perceptions of the work environment emerge (Woodley et al., 2019).

To develop our theoretical framework within the multilevel emergence framework, we draw on Conservation of Resources (COR) theory and explain how emotionally demanding interaction patterns deplete members’ resources and degenerate their work environment perceptions (Hobfoll et al., 2018; Hood et al., 2016). To this end, we focus on climate for autonomy, which refers to the extent to which members experience volition, choice, and psychological freedom in their work while working in the team environment (Liu et al., 2011). We then resort to prior studies that extend the Self-Determination Theory (SDT) to the group level and propose that low autonomy climate in teams with negative affectivity is linked to similarly low levels of team creativity (Deci and Ryan, 2000; Grenier et al., 2024).

To further advance this framework, we turn to the role of leaders as managers of affective dynamics in teams (Madrid et al., 2016). Although prior research has acknowledged the importance of emotions and moods in shaping team functioning (Barsade and Gibson, 2007), relatively little is known about how leaders actively shape these processes. In emotionally challenging environments, such as teams composed of members high in negative affectivity, leaders’ management of affective cues can fundamentally reshape the team’s emotional tone and work climate. To this end, we focus on leader emotion management behavior (LEMB), defined as leaders’ intentional efforts to recognize, regulate, and respond constructively to emotional dynamics within the team (Song et al., 2020). We propose that LEMB plays a pivotal moderating role by influencing how affective cues are interpreted and experienced by team members, thereby attenuating the negative relationship between group negative affectivity and autonomy climate. By considering leaders’ affective intervention, this study extends the group affect literature beyond member-driven processes and highlights the interactive nature of affective dynamics between leaders and team members.

In summary, we propose a conceptual framework in which group negative affectivity is indirectly and negatively related to team creativity through its negative effect on climate for autonomy, with LEMB serving as a critical moderator. This study offers several contributions. First, we shift the focus from transient affective states (e.g., affective tone) to the affective composition of members’ trait affectivity, thereby highlighting the role of stable dispositional inputs in shaping team dynamics. Second, we explicate how these affective inputs translate into team outcomes by identifying climate for autonomy as a key mediating mechanism linking affective composition to team creativity. Third, we advance understanding of leadership by demonstrating that LEMB functions as a boundary condition that shapes how group negative affectivity shapes team climate for autonomy. Finally, we provide empirical support for these arguments using multilevel, multisource field data.

## Hypothesis development

**Figure 1:**
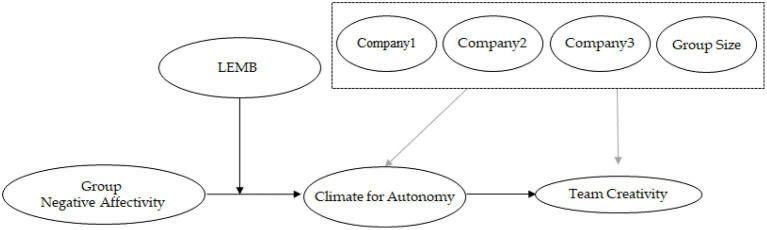
Research model.

Drawing on COR theory (Hobfoll et al., 2018) and SDT (Deci and Ryan, 2000), we develop a group-level conceptual framework to examine the implications of members’ trait negative affectivity for work teams. From a COR perspective, emotionally demanding interpersonal environments characterized by high negative affectivity are likely to deplete members’ cognitive and emotional resources. In addition, SDT highlights autonomy as a central motivational driver of intrinsic engagement and creative performance (Deci and Ryan, 2000; Gagné and Deci, 2005). Based on these perspectives, we argue that teams with high levels of member negative affectivity are more prone to developing dysfunctional climates and experiencing lower creative performance. However, these risks can be mitigated when leaders effectively manage team emotions. Figure 1 presents an overview of the proposed research model. Below, we provide detailed theoretical justifications for each relationship in the proposed model.

Teams characterized by high levels of negative affectivity are more likely to engage in emotionally aversive interactions, such as tension-laden discussions, harsh criticism, and emotional withdrawal (Hood et al., 2016). From the COR perspective, such emotionally demanding environments are likely to deplete members’ resources, thereby undermining the development of a supportive motivational climate. In particular, contexts marked by criticism, defensiveness, and withdrawal (Liu et al., 2011) may lead members to adopt more cautious and constrained behavioral patterns, reducing proactive engagement and self-directed action (Fay and Sonnentag, 2012). Consistent with SDT, these dynamics may inhibit the emergence of an autonomy-supportive climate, as such environments fail to foster volition, psychological safety, and intrinsic motivation (Deci and Ryan, 2000; Gagné and Deci, 2005). Accordingly, we propose the following relationship:

*H1:* Group negative affectivity is negatively related to climate for autonomy.

As a core component of SDT, autonomy is widely recognized as a critical driver of creative engagement and performance, as it supports exploration, persistence, and risk taking in problem solving (Amabile and Pratt, 2016). Building on recent advances in SDT (Grenier et al., 2024), we extend this perspective from its traditional individual-level focus to the team level. Whereas SDT has primarily explained how autonomy-supportive contexts foster individuals’ need satisfaction and autonomous motivation (Deci and Ryan, 2000), emerging work suggests that these motivational processes can also manifest as shared, emergent states within teams (Grenier et al., 2024). Accordingly, we conceptualize climate for autonomy as a shared contextual condition that captures the perceived support for self-direction and choice in the team context (Deci and Ryan, 2000). When team members share a strong sense of autonomy, they are more likely to express ideas, take initiative, and engage in collaborative experimentation (Li et al., 2025; Zhang et al., 2017). In contrast, when autonomy is constrained, team members are more likely to adopt cautious and avoidance-oriented behaviors, limiting their engagement in creative processes (Demerouti et al., 2024). Thus, we propose the following hypothesis:

*H2:* Climate for autonomy is positively related to team creativity.

Building on the above arguments, we further propose that climate for autonomy represents a key mechanism linking group negative affectivity to team creativity. Teams composed of members high in negative affectivity are more likely to experience emotionally demanding interactions characterized by tension, conflict, and emotional strain (e.g., Tsai et al., 2012). Such environments are likely to deplete shared psychological resources and constrain the development of an autonomy-supportive climate. In turn, lower levels of autonomy at the team level are associated with reduced engagement in creativity-relevant processes, such as idea sharing, exploration, and collaborative experimentation (Li et al., 2025; Zhang et al., 2017). Accordingly, we propose that group negative affectivity is indirectly related to team creativity through its negative association with climate for autonomy. This expectation is also consistent with the multilevel emergence framework, which conceptualizes team outcomes as a function of compositional inputs leading to corresponding emergent states (Kozlowski, 2025). Accordingly, we advance the following mediation hypothesis:

*H3:* Group negative affectivity is indirectly related to team creativity via climate for autonomy.

Importantly, the extent to which teams can sustain an autonomy-supportive climate under emotionally demanding conditions may depend on how leaders manage team emotions through LEMB (Song et al., 2020). In the multilevel emergence framework, leaders play a critical role in shaping the interaction patterns through which team climates develop. When negative affectivity is high, team interactions are more likely to become emotionally strained and resource-depleting. In such contexts, in line with COR theory, LEMB may function as a resource-regulating mechanism that alters these interaction patterns by reframing negative experiences, diffusing interpersonal tension, and providing empathic support (Hobfoll et al., 2018). Through these behaviors, leaders can reduce perceived threat and emotional strain, enabling team members to conserve psychological resources and maintain team conditions supportive of autonomy (Hood et al., 2016). Accordingly, LEMB may weaken the negative association between group negative affectivity and climate for autonomy. Therefore, we propose:

*H4:* LEMB moderates the relationship between group negative affectivity and climate for autonomy such that the negative relationship is weaker when LEMB is high.

Taken together, this reasoning suggests that leaders’ affective engagement in the form of LEMB can shape not only team climate but also the broader process through which group negative affectivity is associated with team creativity. Specifically, by weakening the negative implications of group negative affectivity for autonomy climate, LEMB is expected to attenuate the indirect association between group negative affectivity and team creativity. Accordingly, we propose a moderated mediation model in which the indirect relationship between group negative affectivity and team creativity via climate for autonomy varies as a function of LEMB. Thus, the following hypothesis is proposed:

*H5:* LEMB moderates the indirect relationship between group negative affectivity and team creativity mediated by climate for autonomy such that the indirect relationship is weaker when LEMB is high.

## Methods

### Research setting and participants

We collected data from four Korean companies across diverse industries, including semiconductor equipment manufacturing, display technology, vacuum technology, and financial sectors. Participants were nested within 66 work teams drawn from departments such as R&D, sales, human resources, production, and quality control. Surveys were distributed to both team members and their immediate leaders. After removing incomplete or unmatched responses, the final sample consisted of 293 team members and 66 leaders across 66 teams. Team members had an average age of 33.03 years (SD = 5.13) and an average organizational tenure of 4.63 years (SD = 3.70); 12.6% were women. Leaders had an average age of 41.19 years (SD = 4.18) and an average tenure of 10.22 years (SD = 5.84); 3% were women.

### Procedure

We collected multi-source field survey data to reduce common method bias. To obtain access to participants, we contacted human resource (HR) departments in the participating companies and requested their cooperation in administering the survey. With the assistance of HR managers, employees were invited to participate in the study on a voluntary basis. Surveys were distributed to both team members and their immediate leaders. Team members reported their trait negative affectivity, climate for autonomy, and demographic information, whereas leaders evaluated team-level creativity. This study was conducted in accordance with institutional and national research guidelines. Informed consent was obtained from all participants prior to data collection, and participation was voluntary and anonymous. The sample of 66 teams is consistent with prior team-level research and falls within commonly recommended ranges for stable estimation in multilevel and team-based analyses (Bliese, 2000; Hox, 2010; Mathieu et al., 2008).

### Measures

Measurement items were drawn from previously published and validated sources. All scales were originally developed in English and were translated into Korean using a standard translation–back-translation procedure (Brislin, 1980). All items were rated on a 6-point Likert scale (1 = *strongly disagree*, 6 = *strongly agree*).

#### Group negative affectivity

Consistent with a compositional view of group affectivity, group negative affectivity was measured by aggregating individual trait negative affectivity scores. Group-level negative affectivity based on members’ affective trait was assessed using the 10-item negative affect subscale (*α* = 0.92) adopted from the Positive and Negative Affect Schedule (PANAS; Watson et al., 1988). Employees rated how often they generally feel emotions such as distressed, irritable, nervous, and afraid. Aggregated mean scores of members’ individual trait affectivity were used to represent group-level affectivity.

#### Climate for autonomy

Climate for autonomy was measured with a 3-item scale (*α* = 0.79) adapted from Weber et al. (2002), assessing team members’ sense of volition in expressing opinions and making decisions (e.g., “In this team, I feel free and choiceful as I participate” and “I feel wholehearted (as opposed to feeling controlled or pressured) as I do things for this team”). Individual scores were aggregated to the team level.

#### Leader emotion management behavior (LEMB)

LEMB was measured using a 4-item scale developed by Kaplan et al. (2012), evaluating the extent to which leaders manage and respond to emotional dynamics in the team (e.g., “Our team leader effectively manages group conflict” and “Our team leader identifies ostracized members and attempts to reincorporate them into the group,” α = 0.87). Items were rated by employees and aggregated to the team level.

#### Team creativity

Leaders assessed team creativity using a 4-item scale from Zhou and George (2001) (e.g., “This team often generates novel and useful ideas,” α = 0.88).

#### Control variables

At the group level, control variables included company affiliation and team size to isolate the unique influence of negative affectivity.

To reduce common method bias, data were collected from both team members and team leaders, and aggregation procedures were used for group-level variables. Harman’s single factor test also showed no general factor. In addition, the use of multi-source data reduces the likelihood of common method bias.

### Justification for aggregating individual-level reports to the group level

To justify the aggregation of individual-level responses to the group level, we assessed within-group agreement and between-group variability using three indices: interrater agreement (rwg; James et al., 1984), intraclass correlations (ICC[1], ICC[2]; Bliese, 2000), and Eta-squared (η^2^). These statistics provide a basis for evaluating the appropriateness of treating constructs as group-level variables.

For climate for autonomy, the mean rwg across teams was 0.61, with ICC(1) = 0.20 and ICC(2) = 0.46, and η^2^ = 0.35. These values indicate acceptable within-group agreement and meaningful between-group variance (Chen et al., 2005). η^2^ suggested that 35% of the variance in climate for autonomy was attributable to group membership, supporting aggregation at the group level (LeBreton and Senter, 2008). For LEMB, the mean rwg was 0.97, ICC(1) = 0.23, ICC(2) = 0.51, and η^2^ = 0.37. These strong values across all indices provide robust support for aggregating LEMB to the group level.

For group negative affectivity, the aggregation statistics are conceptually less central for justifying its aggregation because it represents a composition construct reflecting the average of individual members’ dispositional traits rather than a shared perceptual or normative state (i.e., additive model versus direct consensus or referent shift model, respectively). These distinctions are consistent with Chan’s (1998) compositional model of aggregation. Nevertheless, we report these indices of group negative affectivity for completeness. The mean rwg was 0.67, ICC(1) was 0.05, ICC(2) was 0.15, and η^2^ was 0.25, indicating modest within-team similarity and some between-team variability. These patterns are consistent with prior multilevel research, which distinguishes compositional constructs from shared perceptual constructs in terms of aggregation requirements.

### Analytic strategy

We tested the hypothesized moderated mediation model using Hayes’ PROCESS macro (Model 7; Hayes, 2013) with 10,000 bootstrap samples. As a robustness check, we additionally conducted a two-level analysis using Mplus 8.10 to explicitly account for the nested structure of the data. In this analysis, group negative affectivity was modeled as an additive composition construct, operationalized as the cluster mean of individual-level negative affectivity. Climate for autonomy and leader emotion management behavior were specified as between-team components derived from individual-level responses, and team creativity was modeled as a between-level outcome. The multilevel model was estimated using TYPE = TWOLEVEL with robust maximum likelihood estimation (MLR), with focal relationships specified at the between-team level.

All variables involved were mean-centered prior to analysis. Specifically, team-level predictors were grand-mean centered before constructing interaction terms. Group-level control variables, i.e., company (dummy-coded) and group size, were included in all analyses. PROCESS was used because all focal variables were aggregated to the team level with adequate aggregation statistics, allowing hypothesis testing at a single level of analysis to evaluate the direct, indirect, and moderated relationships among the current team-level constructs. The multilevel analysis thus serves as a robustness check that the results are not an artifact of single-level estimation but remain consistent when accounting for data nesting.

## Results

### Descriptive statistics and correlations

Table 1 presents the descriptive statistics and intercorrelations among all study variables. Group negative affectivity was negatively correlated with climate for autonomy (*r* = −0.36, *p* < 0.01), which in turn was positively related to team creativity (*r* = 0.24, *p* < 0.05). These correlations are presented for descriptive purposes only and do not constitute formal tests of the current hypotheses.

**Table 1:** Means, standard deviations, and correlations.

Variables	M	SD	1	2	3	4	5	6	7	8
1. Company 1	0.62	0.49	--							
2. Company 2	0.09	0.29	−0.41**	--						
3. Company 3	0.21	0.41	−0.66**	−0.16	--					
4. Size	4.44	1.82	0.07	−0.02	−0.00	--				
5. Group negative affectivity	2.60	0.42	0.18	−0.10	−0.13	−0.16	--			
6. LEMB	3.67	0.48	0.03	−0.10	−0.07	0.05	−0.28*	--		
7. Climate for autonomy	3.53	0.71	0.09	0.00	0.02	−0.01	−0.36**	0.46**	--	
8. Team creativity	3.94	0.69	0.05	−0.14	−0.01	0.18	−0.11	0.12	0.24*	--

*N* = 66 teams. * *p* < 0.05; ** *p* < 0.01.

### Hypothesis testing

Hypothesis 1 predicted that group-level negative affectivity would be negatively related to the climate for autonomy. As reported in Table 2, Model 2, results from the regression analysis confirmed this negative relationship, showing a significant effect (*b* = −0.57, *p* < 0.01), thereby supporting H1. Hypothesis 2 posited a positive association between climate for autonomy and team creativity. This hypothesis was also supported by the data, with a significant positive effect observed (*b* = 0.32, *p* < 0.05).

**Table 2:** Results of regression analysis predicting climate for autonomy.

Variables	Model 1	Model 2	Model 3
	Coeff. (SE)	Coeff. (SE)	Coeff. (SE)
Constant	3.81*** (0.85)	4.66*** (0.58)	3.05*** (0.30)
Company 1	0.55 (0.34)	0.60† (0.31)	0.78** (0.27)
Company 2	0.52 (0.43)	0.43 (0.39)	0.76* (0.35)
Company 3	0.51 (0.37)	0.48 (0.34)	0.76* (0.30)
Size	−0.01 (0.05)	−0.04 (0.05)	−0.04 (0.04)
Group negative affectivity (GNA)		−0.57** (0.17)	−0.27 (0.16)
LEMB			00.40** (0.11)
GNA * LEMB			0.40* (0.19)

*N* = 66 teams. † *p* < 0.10; * *p* < 0.05; ** *p* < 0.01; *** *p* < 0.001.

Hypothesis 3 proposed that group negative affectivity would be indirectly related to team creativity through climate for autonomy. As shown in the regression results presented in Table 2, Model 2, the direct effect of group negative affectivity on team creativity was not significant (*b* = 0.02, *ns*), whereas climate for autonomy was a significant positive predictor of team creativity (*b* = 0.32, *p* < 0.05), consistent with Hypothesis 2. To formally assess the indirect relationship, a mediation analysis using PROCESS Model 4 with 10,000 bootstrap samples was conducted. The results revealed a significant indirect effect of group negative affectivity on team creativity through autonomy (indirect effect = −0.18, bootstrapped SE = 0.15, 95% CI [−0.65, −0.01]). Because the confidence interval did not include zero, this pattern supports Hypothesis 3, suggesting that group negative affectivity is indirectly related to lower team creativity through reduced climate for autonomy (see Table 3).

**Table 3:** Results of regression analysis predicting team creativity.

Variables	Model 1	Model 2
	Coeff. (SE)	Coeff. (SE)
Constant	2.87** (0.60)	2.86** (0.64)
Company 1	−0.39 (0.38)	−0.41 (0.38)
Company 2	−0.74 (0.48)	−0.76 (0.48)
Company 3	−0.42 (0.41)	−0.43 (0.41)
Size	0.09 (0.05)	0.09 (0.05)
Group negative affectivity		0.02 (0.23)
Climate for autonomy	0.31* (0.14)	0.32* (0.15)

*N* = 66 teams. * *p* < 0.05; ** *p* < 0.01.

**Figure 2:**
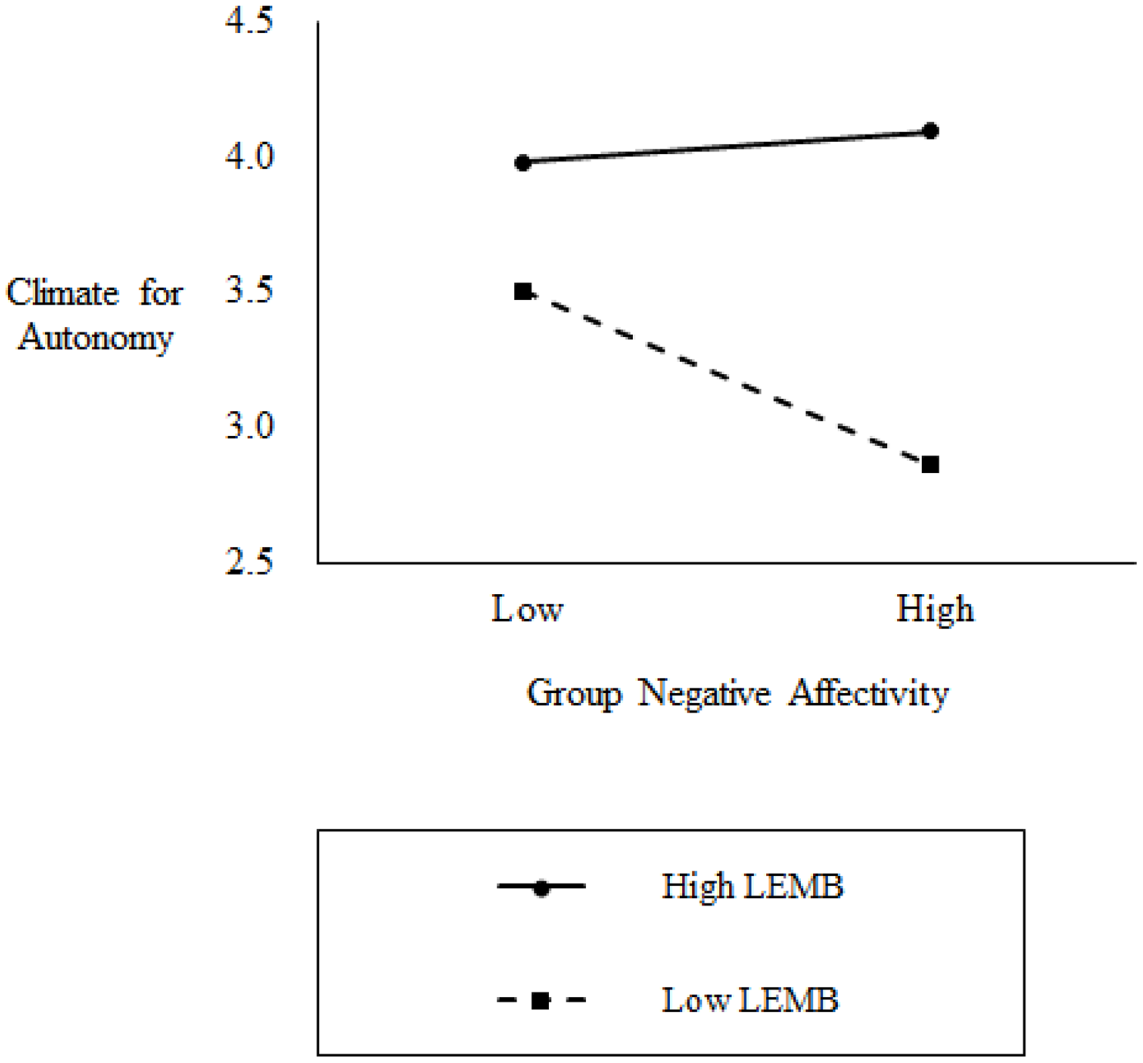
Moderation by leader emotion management behavior.

Hypothesis 4 predicted that LEMB would moderate the relationship between group negative affectivity and climate for autonomy. As shown in Table 2, the interaction term was positive and significant (*b* = 0.42, *p* < 0.05), supporting H4. To further examine this significant interaction, we conducted a simple slopes analysis following Aiken and West (1991). As illustrated in Figure 2, group negative affectivity had a significant negative effect on climate for autonomy when LEMB was low (*b* = −0.67, *p* < 0.05), but no significant effect was observed when LEMB was high (*b* = 0.12, *ns*), consistent with the proposed theoretical expectations for H4.

Hypothesis 5 posited that the indirect relationship between group negative affectivity and team creativity through climate for autonomy would vary depending on the level of LEMB. As reported in Table 4, the conditional indirect effect was significant when LEMB was low (indirect effect = −0.18, 95% CI [−0.61, −0.02]), but it was no longer statistically significant when LEMB was high (indirect effect = 0.01, 95% CI [−0.25, 0.22]). These results support H5.

**Table 4:** Conditional indirect effects of group negative affectivity on team creativity.

Independent variable	Mediator	Dependent variable	Moderator level	Effect	Boot SE	95% bias-corrected CI
Leader emotion management behavior						
Group negative affectivity	Climate for autonomy	Team creativity	Low	−0.18	0.13	(−0.61, −0.02)
			Medium	−0.09	0.10	(−0.47, 0.01)
			High	0.01	0.11	(−0.25, 0.22)

Bootstrap sample = 10,000.

To assess the robustness of these findings, we conducted a two-level analysis that explicitly accounted for individuals nested within teams. The results largely replicated the proposed pattern. Specifically, the interaction between group negative affectivity and leader emotion management behavior on climate for autonomy remained significant (*b* = 0.34, *p* = 0.02), indicating that leader emotion management attenuates the negative effect of group negative affectivity. Climate for autonomy was positively associated with team creativity (*b* = 0.38, *p* = 0.05), consistent with the hypothesized direction. Although the conditional indirect effects did not reach conventional levels of statistical significance, they followed the expected pattern, with stronger negative indirect effects at lower levels of leader emotion management and attenuated effects at higher levels.

### Comparison with alternative models

To assess the robustness and theoretical validity of our hypothesized moderated mediation model, we compared it with three alternative models: (a) a parallel mediation model, (b) a reverse causality model, and (c) a simple mediation model. These models were selected to address potential concerns about competing theoretical relationships and to test whether the interaction between group negative affectivity and LEMB adds incremental explanatory value beyond additive or reversed effects.

All models were estimated using maximum likelihood estimation in Mplus 8.10. Model fit indices, including Akaike Information Criterion (AIC), Comparative Fit Index (CFI), Root Mean Square Error of Approximation (RMSEA), and Standardized Root Mean Square Residual (SRMR), were used to compare overall model performance. A summary of the model fit indices is provided in Table 5.

**Table 5:** Model fit indices for alternative structural models.

Model	AIC	CFI	RMSEA	SRMR
M1: Moderated mediation	282.99	0.92	0.13	0.01
M2: Parallel mediation	438.93	0.55	0.41	0.11
M3: Reverse causality	440.35	0.41	0.27	0.16
M4: Simple mediation	440.35	0.41	0.27	0.16

M1 = hypothesized moderated model; M2 = parallel mediation model specifying independent paths from GNA to climate for autonomy and from LEMB to team creativity; M3 = reverse causality model specifying a path from team creativity to climate for autonomy; M4 = simple mediation model including only the indirect path from GNA to team creativity via climate for autonomy without the interaction term.

The results indicated that the hypothesized moderated mediation model (Model 1) provided a better fit relative to all alternative models. Although the RMSEA value for Model 1 was relatively high (RMSEA = 0.13), we interpret these results in comparative rather than absolute terms. All alternative models exhibited substantially poorer fit across multiple indices. This pattern suggests that, although the absolute fit of the model is not ideal according to conventional standards, the hypothesized model provides a superior representation of the data relative to plausible alternative models. Thus, the results should be interpreted as evidence of relative model superiority rather than as absolute model adequacy. Specifically, Model 1 showed the lowest AIC (282.99), a comparatively higher CFI (0.92), and a low SRMR (0.01), whereas all alternative models produced substantially poorer fit (e.g., CFI < 0.60, RMSEA > 0.25).

In Model 2 (parallel mediation), which specified independent paths from group negative affectivity to climate for autonomy and from LEMB to team creativity, the indirect path through LEMB was not significant (*β* = 0.00, *p* = 1.00), and the overall fit was poor (CFI = 0.55, RMSEA = 0.41). Model 3 (reverse causality), which specified a path from team creativity to climate for autonomy, also failed to account for the data adequately (CFI = 0.41, RMSEA = 0.27). Likewise, Model 4 (simple mediation), which included only the indirect path from group negative affectivity to team creativity via climate for autonomy without the interaction term, demonstrated poor fit across indices. Importantly, the interaction term in Model 1 (GNA × LEMB → Autonomy) was statistically significant (*β* = 0.38, *p* = 0.04), indicating that leaders’ emotion management is associated with differences in how group negative affectivity relates to team climate. These alternative models were examined not only as robustness checks but also to evaluate competing theoretical structures, rather than to establish causal direction, which cannot be inferred from the cross-sectional design.

## Discussion

This study advances understanding of how team-level affective composition is associated with team creativity by drawing on COR theory and SDT in the context of the multilevel emergence framework to understand group-level phenomena. Rather than treating affect as a transient group mood state, we conceptualize group negative affectivity as a relatively stable compositional input based on members’ dispositional affectivity. In doing so, this study responds to recent calls for greater attention to how stable affective characteristics operate at the team level and how they are translated into emergent states and outcomes (Ashkanasy and Dorris, 2017; Barsade and Knight, 2015; Kozlowski and Klein, 2000).

Our analysis shows that group negative affectivity is indirectly related to team creativity through climate for autonomy, which varies depending on leaders’ emotion management behavior. These findings are consistent with the proposed framework, endorsing a process-oriented explanation of how stable team composition inputs translate into differences in team creativity through emergent states such as the climate for autonomy experience by team members.

Importantly, the multilevel robustness analysis yielded a largely consistent pattern of results, particularly with respect to the interaction effect and the conditional structure of the indirect effects. This suggests that the proposed theoretical mechanism is not driven by single-level estimation but remains stable when the nested nature of the data is explicitly modeled. Although some effects were reduced to marginal significance under the multilevel specification, the overall pattern of results remained consistent with the proposed theoretical framework. Below, we discuss the implications and limitations of this study along with further research directions.

### Theoretical implications

This study makes three theoretical contributions. First, it advances research on team affect by shifting attention from transient affective states to affective composition (James et al., 2021). Whereas much prior work has focused on group affective tone or shared emotional states (Baas et al., 2008; Barsade and Knight, 2015; George, 1990), our findings demonstrate that the stable dispositional makeup of team members also plays a critical role in shaping team processes and outcomes. Teams composed of members high in negative affectivity are more prone to emotionally strained interactions (Hood et al., 2016), which are associated with depletion of shared psychological resources and undermine the conditions needed for effective collaboration and creativity (Liu et al., 2020).

Second, this study highlights climate for autonomy as a key motivational mechanism linking affective composition to team creativity. Consistent with SDT (Deci and Ryan, 2000; Nili and Tasavori, 2022), we found that an autonomy-supportive climate is positively related to members’ creative engagement and mediates the relationship between group negative affectivity and team creativity. This result underscores that the consequences of negative affectivity are not limited to direct emotional or interpersonal disruptions; rather, they can unfold through more distal motivational climates that shape how team members approach their work. By identifying autonomy climate as a meaningful pathway, this study integrates affective and motivational perspectives on team creativity.

Third, our results demonstrate that the potential harm of group negative affectivity is not inevitable. The moderating role of LEMB shows that emotionally responsive leadership can prevent the potential loss of resources and protect the team’s motivational climate. When leaders actively manage team emotions by reframing negative experiences, reducing interpersonal tension, and providing empathic support, the negative association between group negative affectivity and climate for autonomy becomes non-significant. In this way, LEMB functions as a resource-protection mechanism that preserves teams’ capacity for autonomous and creative engagement even under emotionally demanding conditions with members’ negative affectivity.

Together, the current findings offer a more contextualized and resource-based account of how group affective composition shapes creative performance in teams. They suggest that negative affectivity should not be viewed solely as a fixed liability but rather as a conditional influence whose effects depend on the availability of team-level resources and leader behaviors. This perspective extends existing work on affective asymmetry in teams (Chi and Lam, 2022) by showing that even strongly negative dispositional inputs can be neutralized when appropriate regulatory mechanisms are in place.

### Practical implications

From a practical perspective, these findings offer actionable guidance for managing teams characterized by high levels of member negative affectivity. In many organizations, managers encounter teams where tension, frustration, or negative emotional tendencies are relatively common. The present results suggest that such conditions should not be viewed as inherently detrimental; rather, their impact depends on how team interactions are managed and how the work environment is structured.

First, because group negative affectivity reflects relatively stable dispositional tendencies, direct attempts to change such traits may not be effective. Prior research indicates that personality represents enduring patterns that persist across situations, even though their expression varies depending on contextual cues (Judge et al., 2008; Mischel, 2004). Accordingly, organizations may benefit more from intervening at the level of team processes and interaction patterns than from attempting to alter individual dispositions. Rather than assuming that teams high in negative affectivity will perform poorly, organizations should assess whether appropriate contextual supports are in place.

Second, the findings highlight the importance of maintaining an autonomy-supportive climate as a critical condition for sustaining team creativity. When team interactions become emotionally strained, members are more likely to behave cautiously and restrictively, thereby limiting idea generation and collaboration. Consistent with SDT and prior research on the work environment and creativity (Amabile and Pratt, 2016), organizations should implement practices that preserve employees’ sense of psychological freedom, such as encouraging open expression of ideas, reducing excessive control, and promoting participative decision-making.

Third, the results underscore the critical role of leaders’ emotion management in regulating team dynamics. Prior work has emphasized that leadership plays a central role in shaping team-level emotional processes (Ashkanasy and Humphrey, 2011). In teams characterized by high negative affectivity, emotionally demanding interactions can accumulate and disrupt collaboration. Leaders who actively manage these dynamics by reframing negative experiences and responding in a supportive and constructive manner can help stabilize interaction patterns and prevent the erosion of an autonomy-supportive climate. Accordingly, organizations need to train leaders to improve their skills and motivation in relation to dealing with emotional issues in the workplace.

Finally, these findings point to a broader shift in managerial focus from attempting to control individual dispositions to actively managing collective interaction processes that sustain autonomy and creativity. By shaping interaction patterns, preserving autonomy-supportive conditions, and managing emotional dynamics, organizations can mitigate the potential downsides of negative affectivity of team members while maintaining team creativity. In addition, organizations may consider the affective composition of teams in team design and staffing decisions.

### Limitations and future research directions

This study has several limitations that suggest directions for future research. First, because group-level emergent states and mediation processes unfold over time, the cross-sectional design limits our ability to directly examine temporal ordering and causal mechanisms. As recent multilevel research has emphasized, cross-sectional designs often capture what has been termed “multilevel statics,” rather than directly observing how emergent processes develop over time (Kozlowski, 2025). Accordingly, the findings should be interpreted as associations that are consistent with the proposed theoretical framework, rather than as evidence of causal or directional relationships. Although alternative model tests provided comparative support for the proposed theoretical structure, longitudinal or experience sampling methods would allow for a more direct examination of how affective composition, climate for autonomy, and creativity evolve over time.

Second, the sample was drawn from Korean organizations, and cultural norms regarding emotion expression and hierarchy may shape these dynamics. In addition, the current sample comprised predominantly male employees, likely reflecting the technology and manufacturing sectors from which the data were drawn. Future research should seek cross-cultural replications with more demographically diverse samples representing various industries to strengthen the generalizability of the findings. Third, although we focused on climate for autonomy as the key mediator, other mechanisms, such as psychological safety, trust, or conflict processes, may also link group affectivity to team creativity. Finally, future research could explore potential nonlinear or interactive effects between positive and negative affectivity, rather than examining negative affectivity in isolation.

## Conclusion

This study advances our understanding of the emotional foundations of team creativity by integrating members’ trait affectivity, motivational climate, and emotion-focused leadership into a unified framework. Although group negative affectivity does not uniformly suppress creativity, its negative effects on climate for autonomy and, by extension, team creativity depend critically on how leaders manage team emotions. In emotionally challenging teams, what leaders do may matter as much as or even more than the affective tendencies of team members themselves.

## Data Availability

The raw data supporting the conclusions of this article will be made available by the authors, without undue reservation.
